# Regulation of Connexins Expression Levels by MicroRNAs, an Update

**DOI:** 10.3389/fphys.2016.00558

**Published:** 2016-11-25

**Authors:** Juan F. Calderón, Mauricio A. Retamal

**Affiliations:** ^1^Facultad de Medicina, Center for Genetics and Genomics, Clínica Alemana Universidad del DesarrolloSantiago, Chile; ^2^Facultad de Medicina, Centro de Fisiología Celular e Integrativa, Clínica Alemana Universidad del DesarrolloSantiago, Chile

**Keywords:** connexins, hemichannels, miRNA, postranscriptional regulation, non-coding RNA, cellular communication

## Abstract

Control of cell-cell coordination and communication is regulated by several factors, including paracrine and autocrine release of biomolecules, and direct exchange of soluble factors between cells through gap junction channels. Additionally, hemichannels also participate in cell-cell coordination through the release of signaling molecules, such as ATP and glutamate. A family of transmembrane proteins named connexins forms both gap junction channels and hemichannels. Because of their importance in cell and tissue coordination, connexins are controlled both by post-translational and post-transcriptional modifications. In recent years, non-coding RNAs have garnered research interest due to their ability to exert post-transcriptional regulation of gene expression. One of the most recent, well-documented control mechanisms of protein synthesis is found through the action of small, single-stranded RNA, called micro RNAs (miRNAs or miRs). Put simply, miRNAs are negative regulators of the expression of a myriad proteins involved in many physiological and pathological processes. This mini review will briefly summarize what is currently known about the action of miRNAs over Cxs expression/function in different organs under some relevant physiological and pathological conditions.

## Introduction

Cell-cell communication and signaling is regulated by exchange of soluble factors between cells through gap junction channels (GJC) (Nielsen et al., 2012). Transmembrane proteins known as connexins (Cxs) form these channels (Vinken, 2015). Interestingly, Cxs not only forms GJCs, but also form another type of channel known as hemichannels (Sáez et al., 2005). When hemichannels open, they allow for the release of bioactive molecules, such as ATP and glutamate to the extracellular media, thus participating in paracrine/autocrine communication (Montero and Orellana, 2015). Also, under certain pathological conditions, hemichannels display a gain of function phenotype, which induces cell malfunctioning or even cell death (Retamal et al., 2015). Cxs are controlled by several post-translational factors, including phosphorylations, oxidations/reductions, and protein-protein interactions, among other mechanisms (Hervé et al., 2012; Pogoda et al., 2016; Retamal et al., 2016). Cxs are also controlled by changes in their expression levels and/or by degradation or stabilization of their corresponding mRNAs (Salat-Canela et al., 2015). A well-documented control mechanism of proteins synthesis is through the action of small single-stranded RNA called micro RNAs (miRNAs–miRs).

## Connexins

Cxs are transmembrane proteins encoded by 21 different genes in humans (Söhl et al., 2005). The canonical structure of Cxs is composed of four transmembrane domains (TM1-4), two extracellular loops (EL1 and EL2), and one intracellular loop (IL1). Both carboxy (C-) and amino (N-) terminal of Cxs face toward cytoplasm (Maeda et al., 2009). The main structural difference between Cxs lies in their C-terminal region, which shows great variability in sequence and length. Because of these differences in protein length, each Cx has been named according to its predicted molecular weight (i.e., Cx43 has a molecular weight of about 43 kDa).

Cxs form two type of channels: GJCs and hemichannels. GJCs are channels composed by longitudinal joining of two hemichannels, which in turn are each composed by six Cxs subunits. Due to its disposition at the plasma membrane, GJCs allow the passive flux of ion and small molecules between cells, while hemichannels allow for the flux of ion and small molecules between the intracellular and extracellular space. These small molecules include ATP, glutamate, glucose, and several second messengers, among others (Retamal et al., 2015). Cell-cell communication and coordination relies on dynamic interchange of signaling molecules between cells; thus, GJCs and hemichannels are key elements of this phenomenon.

GJCs and hemichannels activity is tightly regulated by several mechanisms, including: phosphorylation, redox reactions, cleavages, protein-protein interactions, and changes in pH, among others (Hervé et al., 2012; Pogoda et al., 2016; Retamal et al., 2016). Additionally, Cx levels are controlled post-transcriptionally by mechanisms such as miRNAs, RNA-binding proteins (RBPs), IRES elements, and others (Salat-Canela et al., 2015; Vinken, 2016).

## Biology of miRNAs

**Figure 1:**
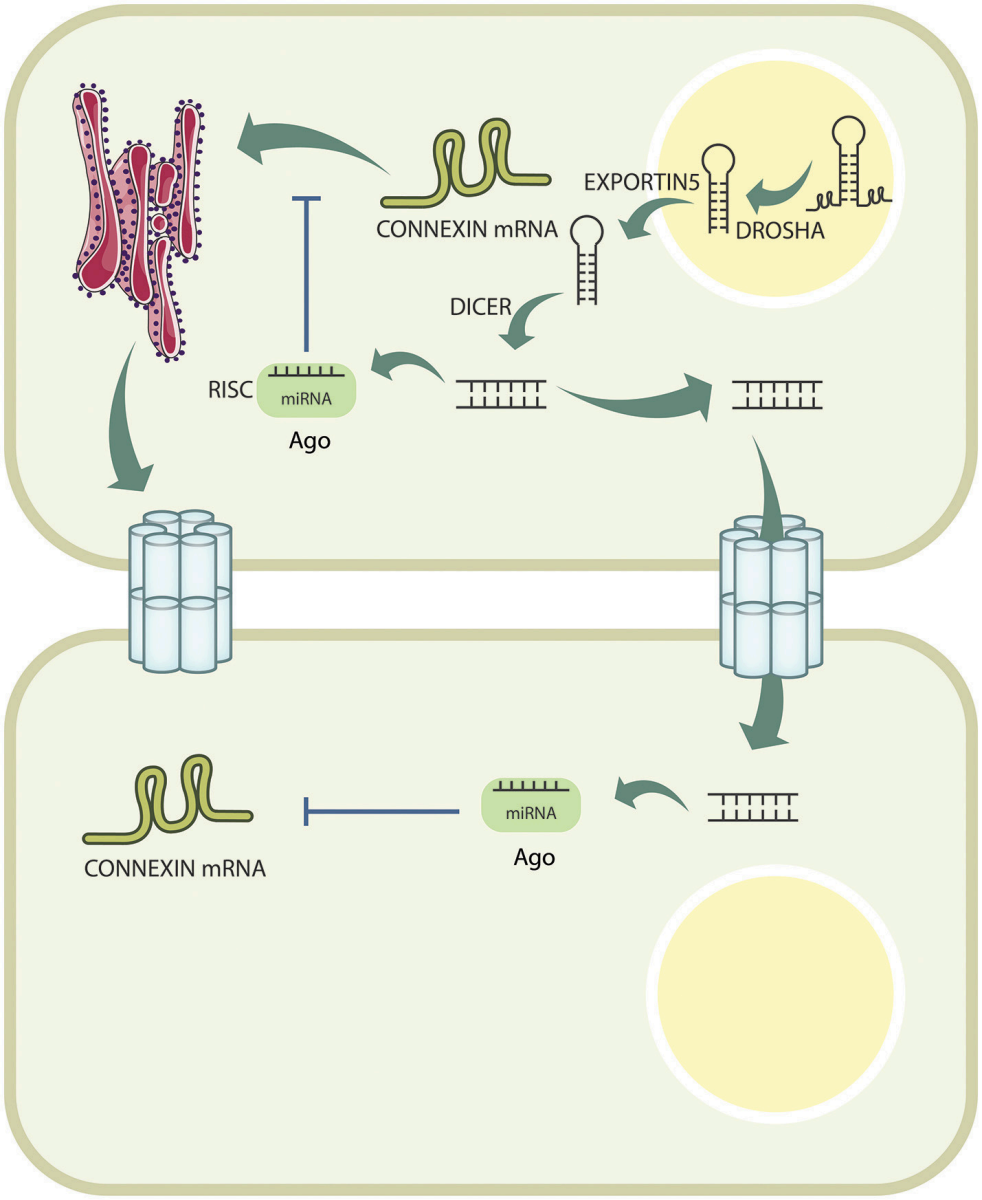
**Connexin expression is actively downregulated by miRNAs intracellularly**. miRNAs are transcribed in the nucleus, and are processed by DROSHA, before being exported to the cytoplasm by EXPORTIN5. Once exported, they are further cleaved by DICER, and loaded onto the AGO protein to form the RISC complex, which will bind to the Cx mRNA, and target it for degradation. Additionally, pre-miRNAs can pass from cell to cell through GJCs, and exert their effect in neighboring cells.

MicroRNAs are a class of 19–25 nucleotide non-coding RNAs, which function in RNA silencing and post-transcriptional regulation of gene expression (Kim, 2005). Most canonical miRNAs are encoded in introns of Pol-II genes; however, others can be located in the minus strand of an exon, although this is an exception rather than a rule (Bartel, 2004). Biogenesis of miRNAs begins with transcription by RNA polymerase II into a single ~300–400 bp transcript (but up to 1 kb in some cases) known as primary RNA transcripts (pri-miRNAs) (Bartel, 2004; Kim, 2005). pri-miRNA usually contains a 5′-CAP structure, and may or may not be polyadenylated on its 3′ end (Ha and Kim, 2014). A pri-miRNA can contain several hairpin structures that leads to the formation of various miRNA-RISC complexes (Lee et al., 2002). Processing of the pri-miRNA transcript initiates with excision of the hairpin structure by the *microprocessor* complex, which includes DROSHA, an RNase III protein, coupled with DGCR8 (Denli et al., 2004). DROSHA acts specifically on dsRNA (like the pri-miRNA) and cleaves off its single stranded portions, capturing the resulting stem-loop structure that is now denominated pre-miRNA (Lee et al., 2003). Subsequently, pre-miRNAs are exported to the cytoplasm through the nuclear pore complex via a Ran-GTP-dependent protein called EXPORTIN5. Once in the cytosol, another RNase called DICER, excises the loop and produce a small RNA duplex (also called miRNA duplex) (Yi et al., 2003; Bohnsack et al., 2004). miRNA duplexes are then loaded onto an Argonaute protein to form the pre- RNA-induced silencing complex (pre-RISC). Subsequently, the so-called “passenger strand” detaches from this complex, completing the formation of the mature RISC complex to target a mRNA for its degradation (Gregory et al., 2005; Matranga et al., 2005). The final configuration of the RISC complex carries the “guide strand” of this miRNA duplex, which is chosen largely due to its relative thermodynamic stability (Kawamata et al., 2009; Winter et al., 2009; Macfarlane and Murphy, 2010). All of these molecular processes are shown in Figure 1.

## Modulation of connexins by miRNAs

miRNAs can significantly downregulate the activity of any given mRNA with a 3′UTR, offering a compatible seed sequence (Bartel, 2009). In the present mini review, we focus on those Cx-miRNAs interactions that may offer potential for investigating new aspects of the pathophysiology of clinically relevant phenotypes.

### Nervous system

There exists a scholarly consensus that cell-cell interactions play a key role in the transition in neuronal activity, which is primarily based on chemical synapses (Moore et al., 2014). Several Cxs are expressed in the brain, including Cx26, Cx32, and Cx43 (Rouach et al., 2002). Cx26 is detected at early stages of the development, while Cx32 and Cx43 are expressed throughout entire brain development and adulthood (Nadarajah et al., 1997). After birth, Cxs play important roles in brain functions, coordinating the activity between neurons and also between glial cells (Pereda, 2014; PosÅuszny, 2014; Decrock et al., 2015; Del Rio et al., 2015). Changes in expression levels and/or channel function formed by several different Cxs have been associated with a number of central nervous system (CNS) disorders. Among these, we can mention X-linked Charcot-Marie-Tooth disease (Bergoffen et al., 1993), traumatic injury of the brain and/or spinal cord (Cronin et al., 2008), hypersynchronous neuronal activity associated with seizures (Seifert et al., 2010; Mylvaganam et al., 2014), and several others (Retamal et al., 2015; Xie et al., 2015). Treatment with a mimetic peptide reduces tissue damage by downregulating gliosis and cytokine release (O'Carroll et al., 2013).

Despite the recognized importance of Cxs for normal brain function and triggering, and/or maintaining of several brains pathologies, to the best of our knowledge, there is no information about the regulation of Cx- mRNA by miRNAs. However, in the peripheral nervous system, when an neuronal damage is generated (i.e., induced by chronic constriction), the level of miR-1 is downregulated with a concomitant upregulation of Cx43 within the endoneurium of the sciatic nerve (Neumann et al., 2015). No evidence of Cx43 upregulation has been observed in the neuronal bodies (Neumann et al., 2015).

### Skeletal and smooth muscles

In the cell line C2C12, which is a mouse myoblast cell line, it has been shown that miR-206 promotes muscle differentiation (Kim et al., 2006). During skeletal muscle development, fusion of myoblasts is a mandatory step. There is evidence that this process requires (at least *in vitro*) the presence of Cx43 GJCs. However, after fusion, Cx43 is downregulated by both miR-206 and miR-1 in myocytes *in vitro* (Anderson et al., 2006); therefore, this is a good example in which miRNAs controls the development of cells by controlling the levels of Cx43 under physiological conditions.

As in skeletal muscle cells, miR-1 also controls Cx43 levels in smooth muscle cells. Thus, in overactive bladder, it was shown that MYOCD downregulates Cx43 expression by controlling miR-1 levels, showing that reduction of Cx43 could be a key factor in this pathology (Imamura et al., 2013).

### Bones

Cx43 is the main Cx expressed in osteocytes, and its presence is fundamental for their differentiation (Civitelli, 2008). When miR-206 was experimentally overexpressed during osteoblast differentiation, an inhibition of osteoblast differentiation—and therefore bone formation *in vivo*—was observed (Inose et al., 2009). This phenomenon was strongly associated to Cx43 downregulation (Inose et al., 2009).

### Cardiovascular system

GJs play a key function in propagating action potentials, and the heart is no exception to this principle. Both Cx40 and Cx43 localize along the axis of atrioventricular conduction, including atrioventricular node, atrioventricular bundle and Purkinje fibers suggesting an important role in conducting the impulse (Gourdie et al., 1993). The role of Cxs in the heart is not limited to the electrophysiological mechanism that regulates heart beating; they are also required for normal heart development. Reaume et al had reported that mice which are null for Cx43 display perinatal death (due to malformations of the right ventricular outflow tract) but are not embryonically lethal (Reaume et al., 1995). This suggests functional compensation among Cxs, a phenomenon that could occur in other tissues and that offers a potential avenue for therapeutic approaches that require further exploration.

As mentioned, miR-1 is involved in downregulation of Cx43 in skeletal muscle development (Anderson et al., 2006). In the heart, miR-1 overexpression has been associated with the appearance of arrhythmias in humans, and this phenomenon is correlated with a reduction in Cx43 expression, which could account for the reduction of the electrical conduction velocity (Yang et al., 2007). Zhang et al. previously observed that when neonatal cardiomyocytes were exposed to an atmosphere with 2% oxygen for 24 h, showed an overexpression of miR-1, and a reduction in Cx43 levels. However, the application of tanshinone IIA (a fat-soluble ingredient of Danshen) to hypoxic cardiomyocytes reduced the expression of miR-1, and restored Cx43 levels, suggesting that tanshinone IIA could play a role in cardiomyocytes protection from ischemic and hypoxic injury (Zhang et al., 2010). It has been previously shown that miR-1 modulates Cx43 levels in response to viral myocarditis (Xu et al., 2012), atrioventricular block after cardiac ischemia (Zhang et al., 2013), and ventricular hypertrophy induced by heart overload (Curcio et al., 2013). These data strongly support the idea that the muscle-specific miRNA, miR-1, is involved in muscle development, and that its overexpression during adulthood is correlated to heart disease through Cx43 downregulation. In addition to the important role of miR-1, recent evidence demonstrates that when miR-130a is upregulated, it induces a decrease of Cx43 protein levels and, as a consequence, both atrial and ventricular arrhythmias were developed in a mice model (Osbourne et al., 2014). The aforementioned evidence strongly supports the hypothesis that upregulation of miR-1 is directly involved in several cardiac pathologies. One notable exception is Tetralogy of Fallot, a severe congenital heart defect in which miR-1 levels decrease and, as predicted, Cx43 levels increase (Wu et al., 2014). However, it remains unknown why the upregulation of Cx43 contributes to particular heart development defects.

miR-1 is not the sole master switch, controlling Cx43 levels in the heart. On one hand, it has been shown that miR-19 a/b decrease Cx43 levels, and that this change is associated with cardiac arrhythmia observed in a mouse constitutively overexpressing the miR-17-92 cluster in smooth muscle and cardiomyocytes (Danielson et al., 2013). On the other hand, miR-23a is upregulated in the heart in post-menopausal women as a consequence of the reduction in estrogen receptor (E2) (Wang et al., 2015). Upregulation of miR-23a in an ovariectomized rat was associated with a reduction of Cx43 levels, providing evidence that miR-23a mediated the repression of Cx43 in estrogen deficiency induced damage of cardiac gap junctions (Wang et al., 2015).

### Cancer

Significant changes in gene expression patterns that promote rapid cell division are the unifying hallmark of tumorigenesis. Each different type of cancer has a distinctive signature of “driver” mutations, which are recurrent across patients and affect genes that encode key components of the cell cycle machinery (Vogelstein et al., 2013). Different members of the Cx family show abnormal expression levels in tumor tissue samples; notable examples include down regulation of gene expression through promoter hypermethylation of Cx26 in invasive breast cancer (Tan et al., 2002), and Cx36 in colorectal carcinoma (Sirnes et al., 2011). Regulation of Cxs through miRNAs has been well characterized in cancer. For example, in human prostate cancer, upregulation of miR-20a induces a reduction in Cx43 levels (Li et al., 2012). The authors also show that downregulation of miR-20a inhibitor (LentimiRa-Off-has-miR-20a Vector) suppresses the proliferation of MDA-PCa-2b cells, both *in vivo* and *in vitro*, and inhibits tumor growth *in vivo* (Li et al., 2012). Likewise, in glioblastoma multiforme, it was observed that downregulation of Cx43 by miR-221/222 is implicated in invasiveness and disease progression (Hao et al., 2012). The level of downregulation has been associated with the degree of malignancy (Hao et al., 2012; Ye et al., 2016). Therefore, when U251 human glioblastoma cells were transfected with antisense oligonucleotides against miR-221/222, Cx43 expression was upregulated, and cellular communication through GJCs was restored (Hao et al., 2012). Similar results were observed when miR-125b was overexpressed (Jin et al., 2013). However, abnormal downregulation of Cx43 by miR-221/222 and by miR-125b has also been observed in astrocytoma (Ciafrè et al., 2005; Conti et al., 2009; Jin et al., 2013).

In nasopharyngeal carcinoma associated with the Epstein-Barr virus, downregulation of miR-218 has been consistently observed (Alajez et al., 2011). Interestingly, this study confirmed that miR-218 targets Cx43 mRNA, and that overexpression of miR-218 induced cell death in C666-1 cell line, which is derived from nasopharyngeal carcinoma (Alajez et al., 2011). However, these results contradict previous results, which demonstrated that Cx43 is downregulated in nasopharyngeal carcinoma (Shen et al., 2002; Xiang et al., 2002; Yi et al., 2007).

In breast cancer cell line MDA-MB-231, transfection of hsa-miR-206 decrease Cx43 levels, which was correlated with a decrease of proliferation rate and cell migration (Fu et al., 2013). Accordingly, higher levels of miR-206 in lymph nodes-negative groups was found when compared to lymph nodes-positive groups (Fu et al., 2013). Thus, at least in breast cancer cells, downregulation of Cx43 may result in a decrease of proliferation and invasion.

An interesting potential avenue of research is to better understand whether changes in expression levels of Cxs in different types of cancer are partially or fully mediated by miRNAs. Therefore, biologically accurate models are required in order to dissect the mechanism that underlies promotion of invasiveness and worsens the clinical course of different forms of cancer.

## Conclusions

This review revisits insurmountable evidence of the relevant role of Cxs in health and disease. In addition to this, we have discussed the most recent findings in microRNA-mediated regulation of Cxs for several muscle and skeletal disorders as well as rhythm-associated and structural heart defects, and several types of cancer. Table 1 contains a detailed list of publications with functional evidence for regulation of Cxs levels by miRNAs. Each microRNA-Cx regulatory relationship can be a potential therapeutic target with clinical implications; thus, the relevance of understanding this mechanism in the context of health and disease. Many challenges lie in testing the functional effect of manipulating Cx levels by repressing or overexpressing their target microRNAs for other diseases; but as additional evidence is found, more innovative therapeutic approaches will be possible.

**Table 1:** **Detailed list of miRNAs predicted to target the different connexin genes, as demonstrated in the associated reference**.

**Gene**	**Common name**	**Associated miRNA**	**References**
GJA1	Cx43	hsa-miR-206	Anderson et al., 2006
		hsa-miR-218-5p	Alajez et al., 2011
		hsa-miR-4266	Lipchina et al., 2011
		hsa-miR-636	
		hsa-miR-648	
		hsa-miR-6888-5p	
		hsa-miR-595	
		hsa-miR-218-5p	
		hsa-miR-651-5p	
		hsa-miR-222-3p	Hao et al., 2012
		hsa-miR-221-3p	
		hsa-miR-20a-5p	Li et al., 2012
		hsa-miR-342-3p	Helwak et al., 2013
		hsa-miR-23b-3p	
		hsa-miR-130a-3p	Osbourne et al., 2014
GJA3	Cx46	hsa-miR-149-5p	Helwak et al., 2013
GJA5	Cx40	hsa-miR-26b-5p	Gennarino et al., 2009
		hsa-miR-1262	Skalsky et al., 2012
		hsa-miR-6842-3p	
		hsa-miR-5787	
		hsa-miR-4505	
		hsa-miR-4430	
		hsa-miR-3652	
		hsa-miR-5589-5p	
		hsa-miR-6776-5p	
		hsa-miR-889-5p	
		hsa-miR-6760-5p	
		hsa-miR-6736-5p	
		hsa-miR-4701-3p	
GJA8	Cx50	hsa-miR-335-5p	Tavazoie et al., 2008
GJB1	Cx32	hsa-miR-335-5p	Tavazoie et al., 2008
		hsa-miR-4763-5p	Chi et al., 2009
		hsa-miR-942-5p	
		hsa-miR-6817-3p	
		hsa-miR-7110-3p	
		hsa-miR-6845-3p	
		hsa-miR-4685-3p	
		hsa-miR-4287	
		hsa-miR-7113-3p	
		hsa-miR-4686	
		hsa-miR-6833-3p	
		hsa-miR-4469	
		hsa-miR-4768-5p	
		hsa-miR-6894-3p	
		hsa-miR-5001-3p	
		hsa-miR-6873-3p	
		hsa-miR-6867-3p	
		hsa-miR-103a-2-5p	
		hsa-miR-1286	Gottwein et al., 2011
		hsa-miR-873-5p	
		hsa-miR-4768-3p	
		hsa-miR-4511	
		hsa-miR-3133	
		hsa-miR-6811-5p	
		hsa-miR-6511b-5p	
		hsa-miR-4722-5p	
		hsa-miR-6813-5p	Whisnant et al., 2013
		hsa-miR-6085	
		hsa-miR-7843-5p	
		hsa-miR-6879-5p	
		hsa-miR-6735-5p	
		hsa-miR-6746-5p	
		hsa-miR-4632-5p	
		hsa-miR-4283	
		hsa-miR-4779	
		hsa-miR-6721-5p	
		hsa-miR-4436b-3p	
		hsa-miR-4763-3p	
		hsa-miR-6891-5p	
		hsa-miR-1207-5p	
		hsa-miR-3173-3p	
		hsa-miR-423-5p	
		hsa-miR-3184-5p	
		hsa-miR-6764-5p	
		hsa-miR-6837-5p	
		hsa-miR-1915-3p	
		hsa-miR-4685-5p	
		hsa-miR-502-5p	Karginov and Hannon, 2013
		hsa-miR-1306-5p	
		hsa-miR-6802-3p	
		hsa-miR-6862-3p	
		hsa-miR-6784-3p	
		hsa-miR-660-3p	
GJB2	Cx26	hsa-miR-335-5p	Tavazoie et al., 2008
		hsa-miR-193b-3p	Chen et al., 2010
		hsa-miR-1295a	Skalsky et al., 2012
		hsa-miR-5704	
		hsa-miR-3142	
		hsa-miR-1245b-5p	
		hsa-miR-4524b-3p	
GJB5	Cx31.2	hsa-miR-335-5p	Tavazoie et al., 2008

## Author contributions

JFC and MAR wrote and edited the manuscript.

### Conflict of interest statement

The authors declare that the research was conducted in the absence of any commercial or financial relationships that could be construed as a potential conflict of interest.
